# Medical Opinion and Movement

**Published:** 1906-06-30

**Authors:** 


### Medical Opinion and Movement.

The address to the students of St. Thomas's Hospital by Dr. William Osler, F.R.S., on the 27th instant, was looked forward to with great interest, and we regret it was delivered too late to enable us to deal with it in the present issue. Anything that Dr. Osler says is sure to be interesting, and to command attention.

Much originality and ability are being brought to bear upon the arrangements for the Toronto meeting of the British Medical Association. Opportunity will be taken to afford European visitors the amplest facilities for visiting everything of interest within the Dominion. Parties of from 18 to 25 will be organised, so that each may have a special sleeping car, an arrangement which will offer the maximum of comfort and convenience to the guests. Dr. Starr and his co-honorary local secretaries are displaying ability and organising power, and names of European visitors should be sent without delay to them, addressed Medical Building, Toronto.

Once again the courts have held that a patient is not entitled to compensation for injuries received, when under treatment, as from a hot-water bottle, unless the clearest evidence of grave negligence is produced. We dealt with the liability of voluntary hospitals for negligence in a leading article in our issue of January 2, 1904. That article, and a previous one published on March 25, 1893, clearly indicate the law on the point. From the standpoint of English law a medical man, whether he be remunerated or not, is personally liable for any negligence or want of skill which he may display in treating a patient. It is, therefore, a marked distinction of the profession in England that actions for compensation are in practice seldom or never brought by patients.

The profession as well as the public cannot fail to be interested in the reports of our special analytical and health commission on tinned and other food-stuffs. Last week the subject was treated generally, and in our present issue appears the first of a series of articles on preserved meat foods prepared in England. We propose to help the public and the profession by reporting upon the chief canned and preserved foods manufactured in this country and by publishing the results of our health commission's inspection of the establishments and manufactories where they are prepared. We hope in this way to enable all to recognise which brands of preserved foods they may safely obtain and use, and that they may so learn what to avoid. If we succeed in our object a vital service will have been rendered to the British people.

An interesting point in regard to dying depositions was raised in the Colonies at a recent trial, when a member of the profession was acquitted of a charge of murder, which mainly rested on the depositions of a deceased woman, a nurse, who had an illegal operation performed, as she deposed, by the practitioner in question. The Judge ruled that the deposition was not admissible on technical grounds. In a previous similar case, where a woman had been convicted by a jury of manslaughter, the first court of appeal annulled the verdict, holding that the deceased's dying depositions were wrongly admitted. The final court of appeal, however, has reversed this decision, holding that the depositions were admissible, and must be taken for what they were worth, and so they upheld the original conviction and sentence. It is not known if the Crown will appeal against the decision of the Judge in the first case referred to.

The present year seems likely to be notable for the activity attributed to those interested in consumption sanatoria. Earnest attempts are being made to induce local authorities to incur large expenditure in the erection of new sanatoria, and district health authorities are being urged with increased frequency to add pulmonary phthisis to the list of diseases notifiable under the Infectious Diseases Act. In pursuance of this crusade members of medical societies are being invited to inspect existing sanatoria so as to impress them with the work of these institutions. The Ochil Hills Sanatorium. Milnathort, was so visited by about forty medical practitioners, when a discussion took place on the notification and treatment of phthisis, which resulted in the passing of a resolution in favour of both these points. We are glad to note that the medical profession as a whole is exhibiting some caution in regard to these questions, and out of forty members present at Milnathort only sixteen voted, so that the decision is not calculated to impress very strongly the local authorities in question.

There can be no doubt that a useful purpose would be served by the issue on authority of a small brochure laying down the course which it is essential for the friends of a patient suffering from tuberculosis to follow on the commencement of the attack. The need of the moment is some clear directions as to how to select and obtain admission to a sanatorium, or to supply sanatorium treatment at home under medical advice, the habits of life which a patient should pursue after the termination of his residence in a sanatorium and throughout life. In German sanatoria there is, no doubt, an excess of feeding, and such patients, when they return to civil life, guard themselves against its continuance. Care must be taken also to find some suitable and regular employment, which will keep both mind and body in a state of activity without involving undue fatigue. Regular exercise, the habit of open-air residence, and a wholesome recognition of the strict limitations of any risk of re-infection, are other essentials to continuous good health. What is wanted is to create a wholesome and rational medical public opinion in regard to tuberculosis and the cure and care of those affected by this disease.

